# Targeting STAT5A via CRISPR/Cas9 restores TKI sensitivity in resistant chronic myeloid leukemia cells

**DOI:** 10.1007/s12032-026-03295-6

**Published:** 2026-04-25

**Authors:** Besne Çelik, Yağmur Kiraz, Yaren Şahin, Burçin Tezcanlı Kaymaz

**Affiliations:** 1 Department of Medical Biology, Faculty of Medicine, Ege University, İzmir, Türkiye; 2 Department of Genetics and Bioengineering, Faculty of Engineering, İzmir University of Economics, İzmir, Türkiye

**Keywords:** CRISPR/Cas9, STAT5A, Chronic Myeloid Leukemia, TKI resistance, Apoptosis, Pathway enrichment

## Abstract

Therapeutic resistance to tyrosine kinase inhibitors (TKIs) remains a major challenge in the clinical management of chronic myeloid leukemia (CML). The transcription factor STAT5A, a principal downstream effector of BCR::ABL1, has emerged as a key transcriptional regulator implicated in the development of TKI resistance. This study aims to functionally validate the role of STAT5A in TKI-resistant CML by employing CRISPR/Cas9-mediated gene knockout and assessing the downstream molecular and phenotypic alterations. We hypothesized that selective disruption of STAT5A would restore apoptotic sensitivity and TKI responsiveness in resistant CML models. Additionally, we sought to integrate bioinformatic transcriptional network analyses to confirm whether STAT5A directly regulates the genes modulated by its deletion, thus reinforcing its mechanistic relevance as a therapeutic target. STAT5A was knocked out using CRISPR/Cas9 in K562 cells and their TKI-resistant derivatives (K562/Ima-Res, K562/Pon-Res). Western blot analysis confirmed effective depletion of STAT5A protein following CRISPR/Cas9 editing, validating that the observed phenotypic and transcriptional changes were attributable to successful STAT5A knockout. Post-editing, XTT assays were performed to assess cell viability, followed by Annexin V/PI staining for apoptosis and PI-based flow cytometry for cell cycle analysis. RT-qPCR was used to quantify the expression of key genes involved in the JAK/STAT pathway (JAK2, STAT3, CISH) and apoptosis/DNA damage responses (TP53, ATM, CASP3, CASP8). In silico analyses were conducted using TRRUST and Harmonizome/ChEA3 to confirm whether the genes modulated by STAT5A deletion were direct transcriptional targets. For additional validation, expression matrices from GSE207627 and GSE208314 were reanalyzed to confirm STAT5A-centered pathway alterations in resistant CML datasets. STAT5A knockout significantly reduced cell viability and induced apoptosis across all CML cell models, accompanied by G0/G1 cell cycle arrest. RT-qPCR revealed altered expression of both JAK/STAT components (JAK2, STAT3, CISH) and apoptosis-related genes (TP53, ATM, CASP3, CASP8). Transcriptional target analysis confirmed that several of these genes—such as CDKN2B, BCL2L1, and CCND1—are direct STAT5A targets, reinforcing the functional consequences of STAT5A loss. Integration of these findings suggests that STAT5A knockout reprograms both intrinsic (CASP3, TP53, ATM) and extrinsic (CASP8, BCL2L1) apoptotic pathways, thereby restoring chemosensitivity. CISH dysregulation further suggested compensatory feedback within the signaling network. CRISPR/Cas9-mediated STAT5A disruption effectively reverses TKI resistance in CML cells by reprogramming apoptotic and proliferative signaling. These findings identify STAT5A as a mechanistically validated and clinically actionable target, supporting its potential for combination strategies with TKIs or STAT5 inhibitors such as pimozide. Integration of transcriptional network analysis supports the mechanistic basis of these effects. STAT5A emerges as a compelling therapeutic target, meriting further investigation in preclinical models and patient-derived samples to evaluate its translational potential. Future validation in patient-derived CD34⁺ CML models may advance STAT5A-based therapeutic design.

**Supplementary Information:**

The online version contains supplementary material available at 10.1007/s12032-026-03295-6.

## Introduction

Chronic myeloid leukemia (CML) arises from a reciprocal translocation between the BCR gene on chromosome 22 and the ABL1 gene on chromosome 9, resulting in the BCR::ABL1 fusion protein. This oncogene is present in about 95% of CML cases and leads to the formation of fusion proteins such as p210, p190, and p229 [1]. The p210 fusion protein, specifically associated with CML, increases tyrosine kinase activity, promoting uncontrolled cell growth and survival [2]. Tyrosine kinases are key regulators of cellular signaling pathways and, when mutated, can cause persistent cell division, bypassing normal apoptosis mechanisms and contributing to cancer progression [3].

Tyrosine kinase inhibitors (TKIs) such as imatinib target BCR::ABL1 and block this uncontrolled growth, improving prognosis in CML patients [4]. However, resistance to TKIs, including second-generation inhibitors such as nilotinib and dasatinib, remains a major clinical challenge. While third-generation TKIs such as ponatinib can overcome some forms of resistance, BCR::ABL1-independent resistance mechanisms continue to be a significant problem [5]. One mechanism of resistance to TKIs involves the activation of alternative signaling pathways such as SRC, RAS/MAPK, JAK/STAT, and PI3K/AKT. In particular, the JAK/STAT signaling pathway, which regulates cell survival, proliferation, and drug resistance, plays an important role in CML. Activation of STAT proteins, especially STAT3 and STAT5, contributes to leukemia progression and resistance to treatment [6, 7]. STAT5, which exists in two isoforms (STAT5A and STAT5B), is crucial for the survival of CML cells and resistance to imatinib [8]. Inhibition of STAT5A has been shown to induce apoptosis and increase sensitivity to TKIs in both imatinib-sensitive and -resistant CML cells, suggesting that STAT5A is a potential therapeutic target [9].

Recent studies have investigated strategies to overcome TKI resistance by targeting deubiquitinating proteins such as USP1. Inhibition of USP1 reduces BCR::ABL1 levels and impairs leukemic cell survival, and has been studied as a complementary therapeutic approach alongside conventional TKIs [10]. These alternative strategies further underscore the importance of directly targeting resistance mechanisms.

Gene editing technologies such as CRISPR/Cas9 offer a powerful tool to combat treatment resistance in CML. CRISPR/Cas9 is based on the RNA-guided adaptive immune system of bacteria and uses the Cas9 protein to create double-strand breaks at specific sites in the DNA, enabling targeted changes [11]. The system uses guide RNA (gRNA) to direct Cas9 to a specific location in the genome, where it can cut the DNA and initiate repair through mechanisms such as non-homologous end joining (NHEJ) [12]. This technology has proven to be highly efficient and versatile, enabling precise gene knockout (KO) or modification. The CRISPR/Cas9 system has been investigated for its potential to treat diseases such as CML by targeting genes that contribute to drug resistance. For example, knocking down STAT5A with siRNA in CML cells resistant to imatinib has shown promising results, restoring sensitivity to TKIs [9]. By selectively knocking out STAT5A, the CRISPR/Cas9 technique could improve the efficacy of TKIs in resistant CML cases and offer a new therapeutic strategy. Therefore, targeting TKI resistance mechanisms that develop independently of BCR::ABL1 may restore the chemotherapeutic response in CML cells.

This study highlights that, despite advances in TKI therapy, resistance remains a significant challenge in CML. Despite major therapeutic advances with first-, second-, and third-generation TKIs, BCR::ABL1–independent resistance remains one of the most critical unmet needs in CML management. Previous reports have suggested that persistent activation of the STAT5 pathway sustains leukemic survival even under kinase inhibition, yet the specific contribution of STAT5A, distinct from STAT5B, remains insufficiently defined. Emerging evidence indicates that STAT5A not only mediates anti-apoptotic signaling but also transcriptionally controls key regulators of the DNA damage response (TP53, ATM) and caspase-dependent apoptosis (CASP3, CASP8).

These findings collectively point to a central role of STAT5A as a transcriptional node linking survival, proliferation, and apoptotic regulation in CML. Therefore, the present study was designed to functionally dissect the role of STAT5A in TKI resistance by applying CRISPR/Cas9-mediated gene knockout in both sensitive and resistant K562 models, combined with transcriptional network analyses. We hypothesized that selective disruption of STAT5A would reprogram aberrant JAK/STAT signaling, restore apoptotic competence, and ultimately resensitize CML cells to TKIs.

This integrative approach provides not only mechanistic insights into STAT5A-driven resistance but also establishes a foundation for future therapeutic strategies targeting the STAT5A axis in refractory CML.Future directions will focus on validating STAT5A-targeted approaches in primary CD34⁺ CML samples and preclinical xenograft models to establish their translational feasibility.

## Materials and methods

### Microarray data acquisition and differential expression analysis

Expression matrices of GSE207627 and GSE208314 (GPL23159) were retrieved from the Gene Expression Omnibus (GEO) database (https://www.ncbi.nlm.nih.gov/geo/*).* Based on the sample metadata, each dataset was classified into TKI-sensitive (S; parental) and TKI-resistant (R) groups according to the experimental source information. All bioinformatic analyses were performed in RStudio. Preprocessed expression data (Series Matrix files) were imported, log2-transformed where necessary, and analyzed using the limma package. For each dataset, differential expression analysis (DEA) was conducted between resistant and sensitive groups using Linear Models for Microarray Data (LIMMA). Genes with adjusted p-value (FDR) < 0.05 and |log2 fold-change| ≥ 1 were defined as significant. Multiple testing correction was applied through the Benjamini–Hochberg method, as previously reported in DEG-based leukemia studies [13, 14]. A STAT5 signaling-related gene panel (*n* = 12) — including *STAT5A*,* STAT5B*,* JAK2*,* SOCS2*,* CISH*,* MCL1*,* CCND1*,* BCL2L1*,* IL2RA*,* PIM1*,* PIM2*, and *MYC* — was curated from the literature. All analyses were performed in R (v4.3.1) using the limma, ggplot2, and ggrepel packages. Expression distributions were visualized with violin-box hybrid plots using ggplot2 and ggrepel, annotated with p-values and FDR-based significance stars (*p* < 0.05, *p* < 0.01, *p* < 0.001). To compare directionality and consistency between datasets, log2 fold-change bar plots and scatter plots for intersecting genes were generated using dplyr and tidyr.

### Culture conditions and confirmation of resistance

The human CML cell line K562 (catalog no. 89121407; ECACC-European Collection of Cell Cultures) was commercially acquired and maintained in Roswell Park Memorial Institute 1640 complete medium (cat. no. 52400025; Biological Industries) with 10% fetal bovine serum (Cat. No. 11550356; Gibco), 1% 2mM L-glutamine (Cat. No. 03–020-1B; Biological Industries) and 1% penicilin/streptomycin (Cat. No. 03–031-1B; Biological Industries). The cytotoxic response of imatinib- and ponatinib-resistant cell lines K562/1.2 μm imatinib and K562/3nM ponatinib to imatinib and ponatinib treatments was assessed using XTT analysis (Cell Proliferation Kit; catalog number 20–30-1000, Biological Industries). Cells were placed in 96-well plates and the drug was administered at the indicated dose. XTT analysis was performed after 48 h of incubation. Three parallel measurements were performed at 450 nm and measured spectrophotometrically at a reference wavelength of 620 nm in a microplate reader (Thermo Scientific Multiskan FC instrument, Scanning for Multiscan F.C.2.5.1 software; Vantaa, Finland). IC50 values were calculated using CalcuSyn 2.0 software (Biosoft). Each IC₅₀ value represents the mean ± SD from three independent biological replicates, confirming reproducibility of the cytotoxic response.

### CRISPR/Cas9-mediated STAT5A gene editing in parental and TKI-resistant K562 cells

Synthetic sgRNA sequences specific for the STAT5A gene were used for the CML cell line (K562): TrueGuide™ Synthetic sgRNA (Assay ID: CRISPR731236_SGM, catalog number: A35533, Thermo Scientific). TrueGuide™ sgRNA Negative Control, non-targeting 1 (catalog number: A35523, Thermo Scientific) was used as a negative control and TrueGuide™ sgRNA Positive Control, CDK4 (human) (catalog number: A35526, Thermo Scientific) was used as a positive control. Cas9 cleavage was performed using TrueCut™ Cas9 Protein v2 (1 mg/mL, 25 µg, catalog no.: A36497, Thermo Scientific). Transfection was performed using the next generation lipid-based Lipofectamine™ CRISPRMAX™ Cas9 Transfection Reagent (catalog number: CMAX00015, Thermo Scientific). Transfection complexes were prepared in Opti-MEM™ Reduced Serum Medium (catalog number: 31985062, Thermo Scientific) by mixing gRNA and Cas9 protein with Cas9 Plus™ Reagent and then combining with diluted Lipofectamine™ CRISPRMAX™ in a separate tube. The mixture was incubated at room temperature for 10–15 min and then added to the cells at 30–70% confluence. The cells were incubated for 48 h at 37 °C in a humidified 5% CO₂ incubator. Transfection efficiency was determined by measuring gene editing activity in the cells transfected with the positive control (CDK4) using the GeneArt™ Genomic Cleavage Detection Kit (Thermo Scientific).

### Analysis of genomic cleavage

The efficiency of gene editing in genetically modified K562 cells was analyzed by PCR using the GeneArt™ Genomic Cleavage Detection Kit. PCR amplification was performed according to the kit protocol with specific primers for the genes STAT5A F: GATAGGTAGGGCATGGGCAAGG STAT5A R: CTGGATGGTAGGGACCCTCT (271 bp) and CDK4 F: GCACAGACGTCCATCAGCC CDK4 R: GCCGGCCCCAAGGAAGACTGGGAG (557 bp). PCR products were verified by electrophoresis on a 2% agarose gel at 100 V for 1 h. GeneRuler 100 bp Plus DNA Ladder (Thermo Fisher Scientific) was used to determine DNA size. The cleavage assay was performed according to the kit protocol. PCR products were subjected to enzymatic digestion and then analyzed by gel electrophoresis.

### Apoptosis and cell cycle assays

The apoptotic status of STAT5A knockout (KO) and control K562 parental and resistant cells was determined using the Annexin V-FITC Apoptosis Detection Kit (BD Pharmingen, Cat. No.: 556547). During early apoptosis, phosphatidylserine (PS), which is normally located on the inner leaflet of the plasma membrane, translocates to the outer membrane surface. Annexin V labeled with FITC binds to the externalized PS and thus enables the detection of apoptotic cells by flow cytometry. Propidium iodide (PI) staining was used to distinguish early apoptotic (Annexin V positive, PI negative) from late apoptotic/necrotic cells (Annexin V and PI positive). Cells that were negative in both stains were considered viable. Apoptosis analysis was performed in triplicate biological replicates and analyzed using a BD Accuri C6 flow cytometer (BD Biosciences).

The effects of STAT5A knockout on cell cycle distribution in parental and resistant K562 cells and control groups were analyzed using the BD Cycletest Plus DNA Reagent Kit (Becton Dickinson, Cat. No.: 340242). Cells were lysed in a buffer containing sodium citrate, sucrose and DMSO with a non-ionic detergent to permeabilize the membranes. Cytoskeletal proteins were removed by trypsin treatment. RNA was degraded with RNase A and chromatin was stabilized with spermine. DNA was stained with propidium iodide (PI) and fluorescence was measured between 580 and 650 nm to determine DNA content. This allowed quantification of the cells in the G0/G1, S and G2/M phases. For each sample, 1 × 10^6^ cells were analyzed on a BD Accuri C6 flow cytometer (BD Biosciences).

### Isolation of total RNA, cDNA synthesis and qRT-PCR reaction

Cells were collected after incubation and centrifuged to obtain pellets. Isolation of total RNA was performed using the Magna Pure Compact RNA Isolation Kit (Roche) according to the kit protocol with the automated MagnaPure Compact LC system. After adding 100 µl PBS and 100 µl Lysis-binding Buffer to the cell pellet and mixing, the automated isolation procedure was started. The purity and concentration of the isolated RNA were measured by the absorbance ratio at 260/280 nm and 230/260 nm with a Nanodrop device (Thermo Fisher). cDNA synthesis was performed from RNA samples using the OneScript^®^ Plus cDNA Synthesis Kit (Cat. No.G236). First, genomic DNA was eliminated by incubating RNA and Buffer GE at 42 °C, followed by reverse transcription. The reaction mixture was then stored on ice and prepared for real-time PCR. The primer sequences and corresponding genes are detailed in Table 1 and Supplementary Table 1. The synthesized cDNA template was combined with RT2 SYBR Green Mastermix (Cat. No. abm G891) and RT2 qPCR primer assay, and amplification was performed using the LightCycler 480 instrument. qRT-PCR data were analyzed using the 2^-ΔΔCt method. Normalization was performed using *GAPDH* as the internal reference gene to ensure data comparability across conditions.

**Table 1:** Target Genes Used for Quantitative Real-Time PCR (qRT-PCR) Analysis

Related Pathway	Apoptosis	Cell Cycle	JAK/STAT Pathway	Other Genes	STAT5 Target Genes	Oncogene Related Genes	Housekeeping
Genes	*BAX* *BCL2L1 (BCL-xL)* *BCL-2* *CASP3* *CASP8*	*ATM* *TP53*	*STAM* *STAT1* *STAT3* *STAT5A* *STAT5B* *SOCS1* *JAK2*	*BCR*	*CISH* *mTOR*	*ABL1* *RUNX3*	*GAPDH*

### Quantitative visualization and expression normalization

For comparative visualization of gene expression across baseline and TKI-resistant conditions, normalized expression values were computed based on untreated (UT) reference levels. For each gene, absolute expression intensities in STAT5A-knockout (KO) and untreated (UT) K562 models were derived using fold-change scaling, calculated as (2^log₂FC × UT expression). This relative normalization approach was applied to enhance visualization clarity and comparability across datasets without altering directional biological trends. All plots were generated in RStudio (v4.3.1) using ggplot2 and dplyr, and statistical significance was determined using one-way ANOVA with Tukey’s post hoc test (*p* < 0.05, **p < 0.01, **p < 0.001).

### Protein extraction and western blot analysis

For protein expression analysis, whole-cell lysates were prepared using RIPA buffer containing protease inhibitors (Sigma, Cat. No. P8340). Total protein concentrations were determined using the BCA assay (Thermo Scientific). Proteins (30–50 µg) were separated on 12% SDS-PAGE gels and run at 200 V for approximately 50 min using the XCell SureLock Mini-Cell Electrophoresis System (ThermoFisher, Cat. No. EI0001). Separated proteins were transferred to PVDF membranes using the iBlot dry transfer system (Invitrogen Corporation, Carlsbad, CA, USA). Membranes were blocked with 5% non-fat milk in TBST and incubated with primary antibodies against STAT5A (Upstate Cell Signaling Solutions, Cat. No. 06–968) and β-actin (Cell Signaling Technology, Cat. No. 4970), followed by incubation with HRP-conjugated secondary antibodies. Signal detection was performed using enhanced chemiluminescence (ECL). Band intensities were quantified using ImageJ 1.46r software (http://rsbweb.nih.gov/ij/), and data were normalized to β-actin levels [15].

### Statistical analysis

All experiments were performed in triplicate. Data are presented as mean ± standard deviation (SD). Statistical analyses were performed using GraphPad Prism v9.0 and RStudio. Comparisons between two groups were conducted using an unpaired two-tailed Student’s t-test. For comparisons involving multiple groups, one-way analysis of variance (ANOVA) followed by Dunnett’s or Tukey’s post hoc test was applied as appropriate. A p-value < 0.05 was considered statistically significant.

## Results

To comprehensively elucidate the molecular and functional consequences of *STAT5A* disruption in chronic myeloid leukemia (CML), we integrated bioinformatic, genetic, and phenotypic analyses across parental and tyrosine kinase inhibitor (TKI)-resistant K562 models. The results are presented in a multi-layered framework encompassing (i) transcriptomic profiling of the STAT5 signaling network using public CML datasets, (ii) functional validation of CRISPR/Cas9-mediated *STAT5A* knockout, and (iii) downstream phenotypic characterization including cell viability, apoptosis, and cell-cycle modulation. This integrative approach enables a mechanistic dissection of *STAT5A*-dependent resistance pathways and clarifies how targeted gene editing reprograms survival and apoptotic circuits in TKI-refractory CML cells.

### Transcriptomic remodeling of the STAT5 network underlies tyrosine kinase inhibitor resistance in chronic myeloid leukemia

**Fig. 1:**
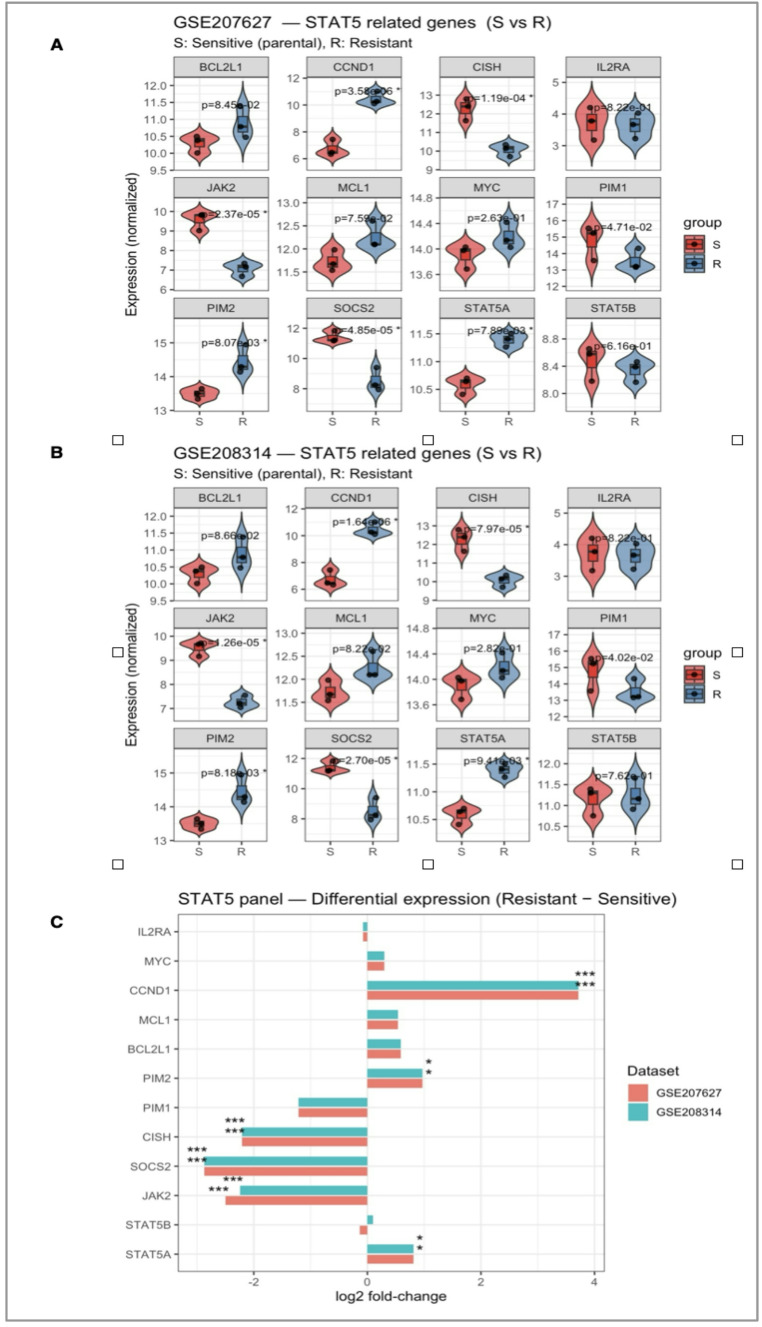
Transcriptomic comparison of STAT5-associated signaling in TKI-sensitive and -resistant CML cells. Differential expression analysis of STAT5-related genes (STAT5A, STAT5B, JAK2, SOCS2, CISH, MCL1, CCND1, BCL2L1, IL2RA, PIM1, PIM2, MYC) across TKI-sensitive (S) and -resistant (R) CML datasets. **A** Expression patterns in GSE207627 (S vs. R). **B** Validation of the same gene panel in GSE208314. **C** Comparative log2 fold-change values of each gene across both datasets (resistant vs. sensitive). A shared color legend is applied for all panels (red = sensitive/parental; blue = resistant). Significance levels: *p* < 0.05 (*), *p* < 0.01 (**), *p* < 0.001 (***)

To explore the molecular basis of tyrosine kinase inhibitor (TKI) resistance, we first examined transcriptomic changes across two independent datasets (GSE207627 and GSE208314). Across two independent datasets (GSE207627 and GSE208314), consistent transcriptional alterations were detected within the STAT5 signaling network (Fig. 1). Both STAT5A and STAT5B, together with the upstream kinase JAK2, were markedly downregulated in resistant clones, suggesting attenuation of canonical STAT5 activity upon chronic TKI exposure. In contrast, MCL1, CCND1, and BCL2L1—genes associated with survival and proliferation—were upregulated, implying compensatory activation of alternative pro-survival pathways. Notably, negative feedback regulators SOCS2 and CISH exhibited concordant suppression, indicating a breakdown in the autoregulatory control of STAT5 signaling.

Collectively, these patterns suggest a transcriptional rewiring from a STAT5A-driven regulatory circuit toward alternative survival mechanisms, providing a mechanistic basis for resistance acquisition. This observation guided subsequent analyses focusing on the functional and prognostic relevance of STAT5A in TKI-resistant CML.

### Baseline viability and cytotoxic response profiles of parental and TKI-resistant K562 cells

**Fig. 2:**
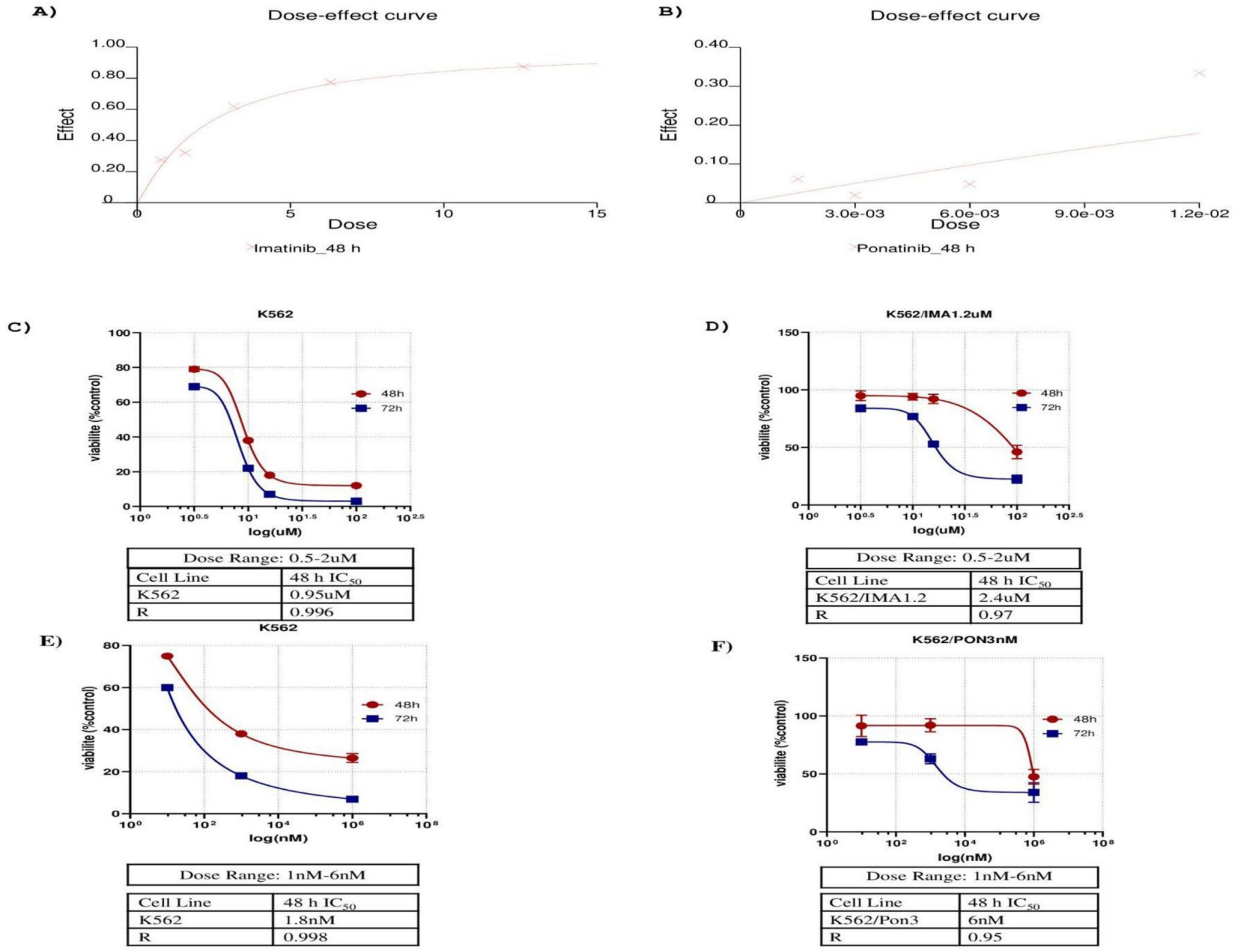
Cytotoxicity profiles of TKI-resistant chronic myeloid leukemia models. **A, B** XTT viability assay results for K562/1.2 µM imatinib-resistant and K562/3 nM ponatinib-resistant cells following 48 h drug exposure. Dose-dependent reductions in cell viability were used to determine IC₅₀ values. Data represent mean ± SD from three independent experiments; IC₅₀ values were calculated using CalcuSyn 2.0 software. **C–F** Dose–response curves of parental and resistant K562 cell lines to TKIs. Parental K562 cells exhibited a marked dose- and time-dependent decrease in viability in response to imatinib (**C**) and ponatinib (**E**). K562/1.2 µM imatinib-resistant cells (**D**) displayed reduced sensitivity to imatinib, as indicated by a rightward shift in the dose–response curve. K562/3 nM ponatinib-resistant cells (**F**) showed diminished responsiveness to ponatinib, with an IC₅₀ exceeding the concentration used for resistance selection. All data are presented as mean ± SD and were analyzed using GraphPad Prism (*n* = 3)

The viability of cultured K562, K562/1.2 µM imatinib-resistant, and K562/3 nM ponatinib-resistant cells was assessed by trypan blue staining. The analysis showed that K562 cells had a viability of 95%, imatinib-resistant cells 92%, and ponatinib-resistant cells 95%. These results indicate that all cell groups had sufficient viability for subsequent experimental studies. To investigate the cytotoxic effects and the response of the resistant cell lines to the drug, an XTT assay was performed. K562/1.2 µM imatinib-resistant cells were treated with 0.5, 1, 1.2, and 2 µM imatinib, while K562/3 nM ponatinib-resistant cells were treated with 1, 3, and 6 nM ponatinib. The data obtained were analyzed using Calcusyn software, and the IC₅₀ values were calculated. After 48 h, the IC₅₀ value of the imatinib-resistant cells was 2.4 µM, while the ponatinib-resistant cells had an IC₅₀ value of 6 nM (Fig. 2A and B).In addition, drug response curves generated for parental and resistant cell lines showed marked differences in sensitivity to TKI treatment (Fig. 2C–F). Parental K562 cells displayed a pronounced dose- and time-dependent reduction in viability after exposure to both imatinib and ponatinib (Fig. 2C and E). In contrast, K562/1.2 µM imatinib-resistant cells showed reduced sensitivity to imatinib, as indicated by a rightward shift in the dose–response curve and increased IC₅₀ values compared to parental cells (Fig. 2D). Similarly, K562/3 nM ponatinib-resistant cells exhibited diminished responsiveness to ponatinib, requiring higher drug concentrations to achieve comparable cytotoxic effects, with an IC₅₀ value exceeding the ponatinib concentration used for resistance selection (Fig. 2F).

### Lipofectamine CRISPRMAX-Mediated Delivery Enables Efficient CRISPR/Cas9 Genome Editing of STAT5A in K562 Cells

**Fig. 3:**
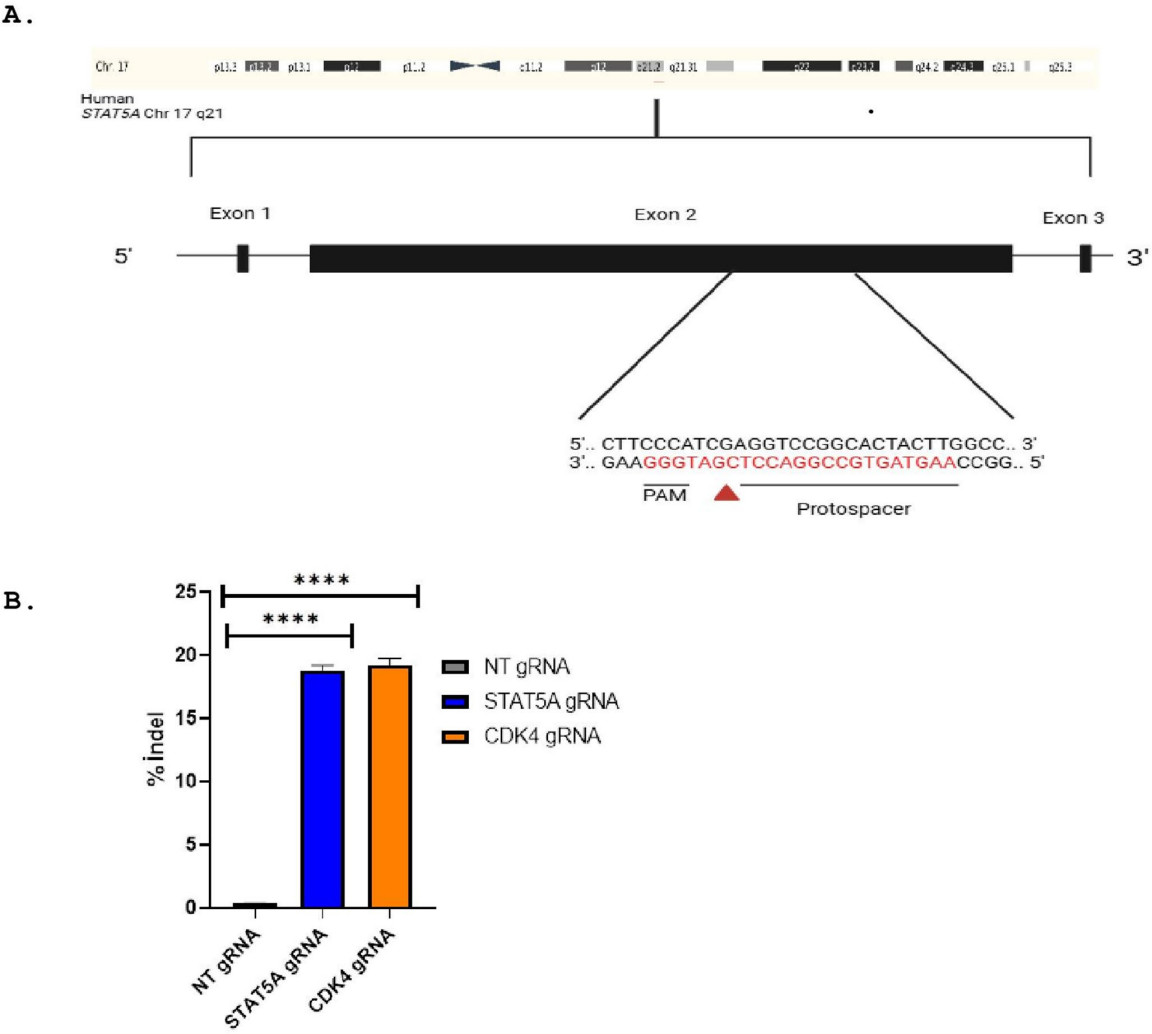
CRISPR/Cas9 targeting of the STAT5A gene and indel efficiency analysis. **A** CRISPR/Cas9 targeting site within exon 2 of the STAT5A gene. The red sequence indicates the gRNA target region, and the triangle denotes the PAM site. **B** Indel frequencies (%) were quantified 48 h post-transfection. Non-targeting gRNA (NT gRNA) was used as a negative control, and CDK4 gRNA served as a positive control for editing efficiency. Data represent mean ± SD from three independent experiments (NT gRNA: 0.38 ± 0.03; STAT5A gRNA: 18.80 ± 0.41; CDK4 gRNA: 19.23 ± 0.55). Statistical significance was determined by one-way ANOVA followed by Dunnett’s multiple comparisons test versus the non-targeting gRNA control (****p < 0.0001). Full, uncropped gel and blot images corresponding to previous panels (C–D) are provided in Supplementary Figures S1–S2

K562 cells (2.5 × 10^5^) were seeded in 6-well plates and transfected with guide RNAs targeting STAT5A, CDK4, and BRD4 along with Cas9 protein using Lipofectamine CRISPRMAX. After 48 h, cells were harvested, and genome editing efficiency was assessed using the GeneArt™ Genomic Cleavage Detection Kit. Gel electrophoresis analysis showed successful cleavage at the target loci. Targeting exon 2 of STAT5A (target sequence: AAGTAGTGGCCGGACCTCGAT, PAM: GGG) resulted in distinct bands at 271 bp and 220 bp. In the positive control (CDK4), cleavage products were observed at 557 bp, 342 bp, and 235 bp. No cleavage or additional bands were detected in the negative control group (non-targeted gRNA) Supplementary Figs. 1 and 2. Indel analysis revealed a cleavage efficiency of 18.8% in STAT5A knockout cells and 19.2% in CDK4 knockout cells **(**Fig. 3B**)**. No significant cleavage was observed for BRD4. These results demonstrate that the CRISPR-Cas9 system can be efficiently introduced into K562 cells with Lipofectamine CRISPRMAX, enabling precise gene silencing at the desired sites (Fig. 3).

**Fig. 4:**
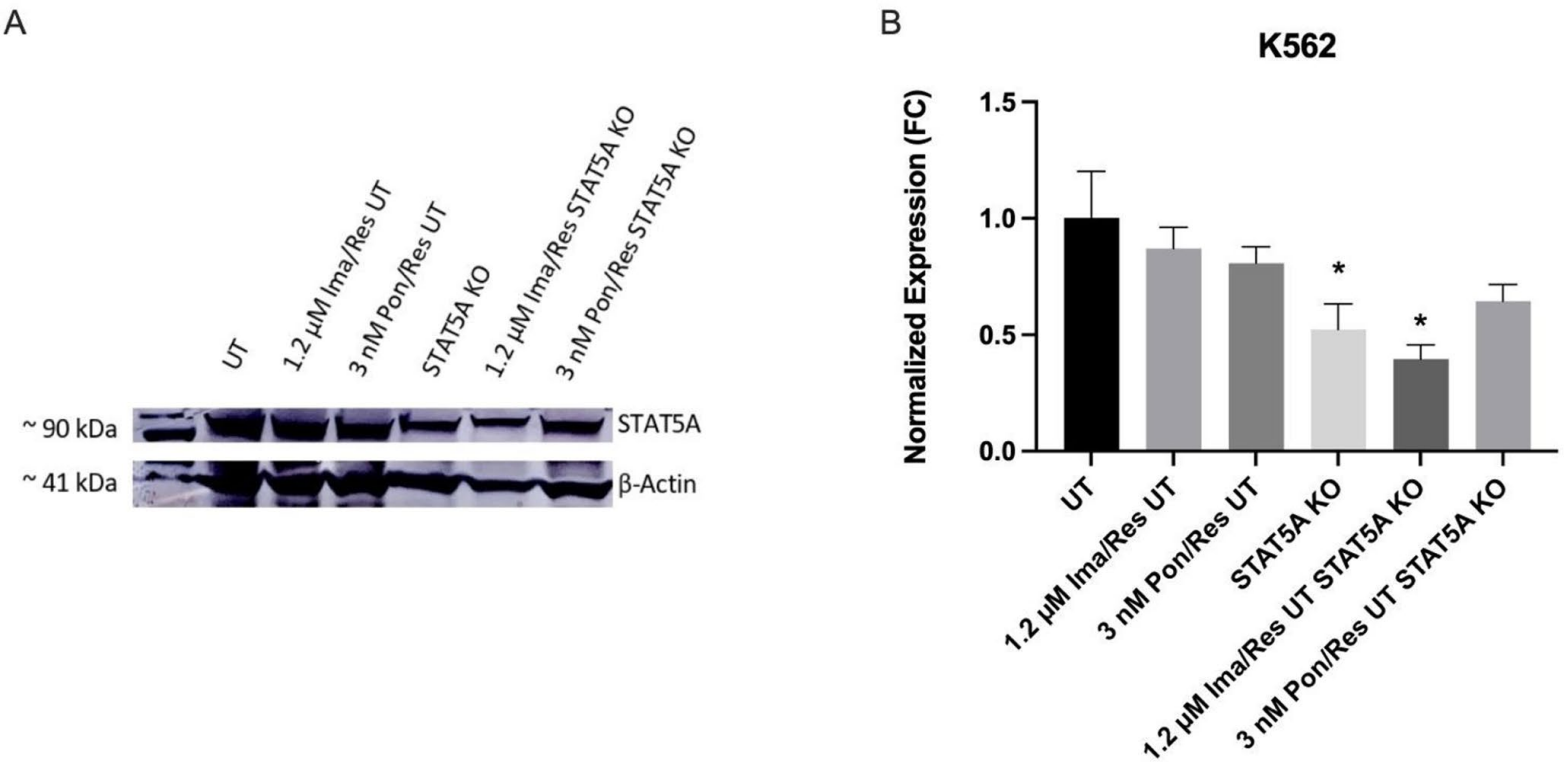
Validation of CRISPR/Cas9-mediated STAT5A knockout at the protein level in K562 cells. **A** Representative Western blot analysis showing STAT5A protein expression (~ 90 kDa) in untreated (UT) K562 cells, imatinib-resistant (1.2 µM Ima/Res UT), ponatinib-resistant (3 nM Pon/Res UT), STAT5A-knockout (STAT5A KO), and STAT5A-knockout resistant cell lines. β-actin (~ 41 kDa) was used as a loading control. **B** Densitometric quantification of STAT5A protein levels normalized to β-actin and expressed as fold change relative to untreated control cells. Data represent mean ± SD from three independent experiments. Statistical significance was determined using one-way ANOVA followed by Tukey’s post hoc test (*p < 0.05)

To confirm that CRISPR/Cas9-mediated genome editing effectively depleted STAT5A at the protein level, Western blot analysis was performed in parental K562 cells and TKI-resistant derivatives after STAT5A targeting. As shown in Fig. 4A, STAT5A-knockout cells exhibited a marked reduction in STAT5A protein expression compared to non-targeting control groups in all cell models. Densitometric analysis (Fig. 4B) further confirmed a consistent decrease in STAT5A protein levels, demonstrating the functional efficiency of the CRISPR/Cas9 editing strategy. β-actin was used as a loading control to ensure equal protein loading across samples. These results indicate that the observed phenotypic and transcriptional effects in subsequent experiments are associated with effective STAT5A depletion.

### CRISPR/Cas9-mediated STAT5A knockout induces apoptosis in K562 and TKI-resistant cells

**Fig. 5:**
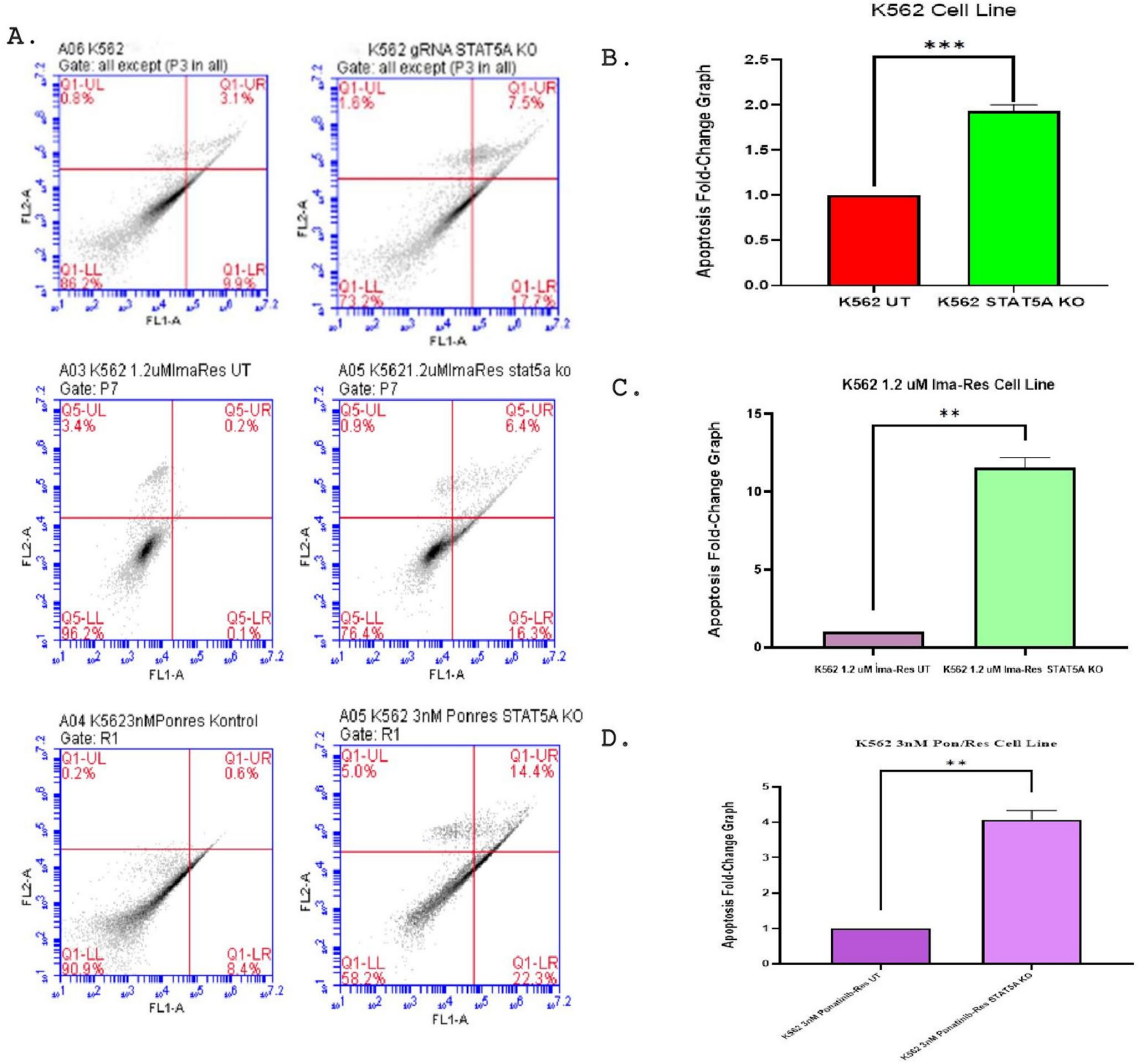
**A** Apoptosis was analyzed 48 h after transfection in K562 control cells, K562 TKI-resistant control cells, and in groups treated with CRISPR/Cas9 for STAT5A knockout (**B**) Fold change in apoptosis levels measured 48 h after transfection in K562 control and STAT5A knockout (KO) cells (mean ± SD 1.93 ± 0.075, *P* = 0.0005). **C** Fold change in apoptosis levels measured 48 h after transfection in K562/1.2 μm Imatinib resistant cells and K562/1.2uMimatinib resistant STAT5A knockout (KO) cells (mean ± SD 11.53 ± 0.66, *P* = 0.011) (**D**) Fold change in apoptosis levels measured 48 h after transfection in K562/3nM ponatinib resistant cells and K562/3nM ponatinib resistant STAT5A knockout (KO) cells (mean ± SD 4.07 ± 0.26, *P* = 0.0014). Statistical significance was determined using an unpaired two-tailed Student’s t-test

STAT5A was knocked out in both K562 control cells and resistant K562 cell lines using CRISPR/Cas9, and apoptotic effects were analyzed 48 h after transfection. In K562 control cells, the apoptosis rate was 9.9% early apoptosis, 3.1% late apoptosis, and 0.8% necrosis, while knocking out STAT5A in K562 cells resulted in 17.7% early apoptosis, 7.5% late apoptosis, and 1.6% necrosis, representing a 1.93-fold increase in apoptosis compared to the control group. In the 1.2 µM imatinib-resistant K562 cell line, STAT5A knockout induced 16.3% early apoptosis, 6.4% late apoptosis, and 0.9% necrosis, corresponding to an 11.35-fold increase in apoptosis compared to the control group. In the 3 nM ponatinib-resistant K562 cells, the control group exhibited 8.4% early apoptosis, 0.6% late apoptosis, and 0.2% necrosis. Knockout of STAT5A increased these rates to 22.3% early apoptosis, 14.4% late apoptosis, and 5% necrosis, a 4.07-fold increase in apoptosis compared to the control group. These results indicate that knockout of STAT5A significantly increases apoptotic processes in both the control group and the resistant K562 cell lines (Fig. 5). This apoptotic enhancement was statistically significant across all biological replicates, supporting the robust pro-apoptotic effect of *STAT5A* depletion.

### STAT5A knockout modulates cell cycle progression in K562 and TKI-resistant K562 cell lines

**Fig. 6:**
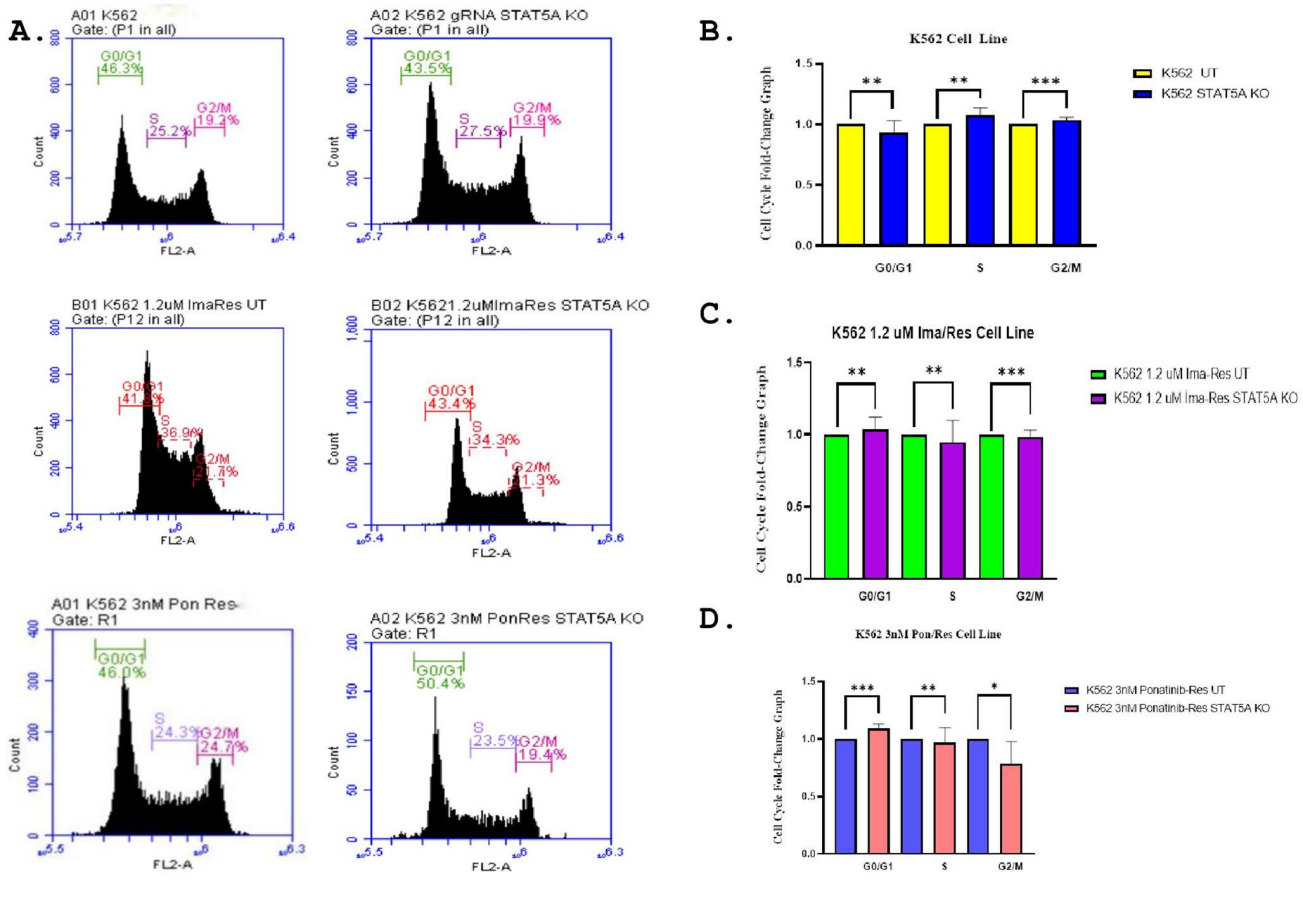
**A** Cell cycle analysis performed 48 h after transfection in K562 control, imatinib-resistant, and ponatinib-resistant cells, as well as in K562 STAT5A knockout and resistant STAT5A knockout cell lines. **B** Fold change graph of cell cycle analysis in K562 control and K562 STAT5A knockout cells (G0/G1 mean ± SD = 0.93 ± 0.095, *P* = 0.0034,* S*: mean ± SD = 1.07 ± 0.060, *P* = 0.0010, G2/M mean ± SD = 1.03 ± 0.02, *P* = 0.0001) (**C**) Fold change graph of cell cycle analysis in K562/1.2 μm imatinib resistant cells and K562/1.2 μm imatinib resistant STAT5A knockout cells (G0/G1 mean ± SD = 1.03 ± 0.085, *P* = 0.0022,* S*: mean ± SD= 0.94 ± 0.15, *P* = 0.0082, G2/M mean ± SD = 0.98 ± 0.05, *P* = 0.0009) (**D**) Fold change graph of cell cycle analysis in K562/3nM ponatinib resistant cells and K562/3nM ponatinib resistant STAT5A knockout cells (G0/G1 mean ± SD = 1.09 ± 0.04, *P* = 0.0004,* S*: mean ± SD= 0.96 ± 0.13, *P* = 0.0060, G2/M mean ± SD = 0.78 ± 0.19, *P* = 0.0189) Statistical significance was determined using an unpaired two-tailed Student’s t-test

In the K562 control cells and the resistant K562 cell lines, STAT5A was knocked out using CRISPR/Cas9, and cell cycle analysis was performed 48 h after transfection. In K562 control cells, the distribution of cell cycle phases was 46.3% G0/G1, 25.2% S, and 19.2% G2/M. After knockout of STAT5A, K562 cells exhibited 43.5% G0/G1, 27.5% S, and 19.9% G2/M, corresponding to a 0.93-fold decrease in G0/G1 and a 1.07-fold increase in S phase compared to control (Fig. 5A-B). In 1.2 µM imatinib-resistant K562 cells, the control distribution was 41.8% G0/G1, 36.9% S, and 21.7% G2/M, while STAT5A knockout resulted in 43.4% G0/G1, 34.3% S, and 21.3% G2/M, corresponding to a 1.04-fold increase in G0/G1 and a 0.95-fold decrease in S phase compared to control (Fig. 6A-C). In 3 nM ponatinib-resistant K562 cells, control cells exhibited 46.0% G0/G1, 24.3% S, and 24.7% G2/M. Knockout of STAT5A resulted in 50.4% G0/G1, 23.5% S, and 19.4% G2/M, corresponding to a 1.09-fold increase in G0/G1 and a 0.78-fold decrease in G2/M compared to control (Fig. 6A-D).These results indicate that knockout of STAT5A affects cell cycle progression in both the K562 control and the imatinib- and ponatinib-resistant cell lines, primarily through an increase in G0/G1 phase and changes in S and G2/M phases (Fig. 6).

### STAT5A knockout modulates the expression of apoptosis and survival genes in K562 and TKI-resistant cells

**Fig. 7:**
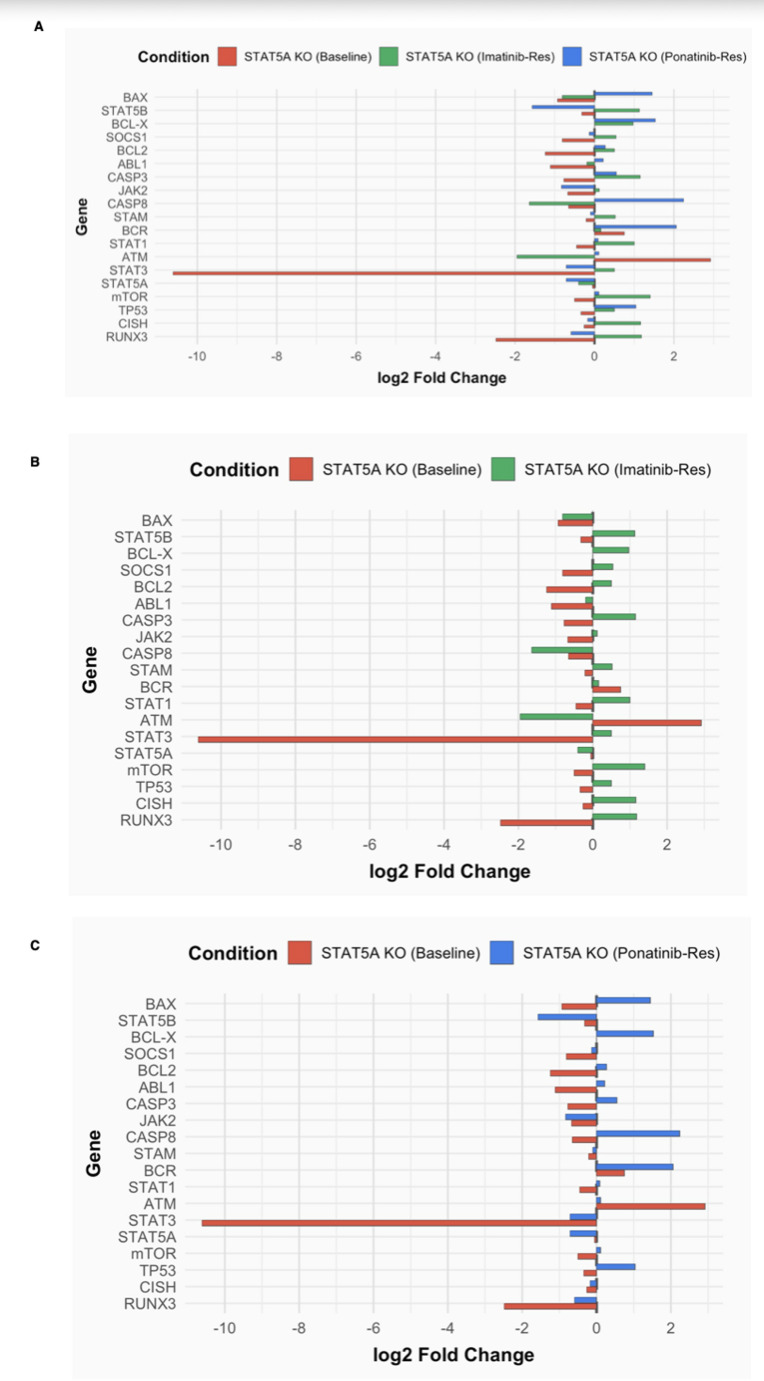
Transcriptional reprogramming following STAT5A knockout in K562 and TKI-resistant models. **A** Comparative log₂ fold change of selected genes across baseline, imatinib-resistant (1.2 µM), and ponatinib (3 nM) conditions in STAT5A-knockout K562 cells (dashed line = 0). **B** Differential expression (log₂ fold change) of apoptosis- and signaling-related genes in STAT5A-knockout K562 cells under baseline and imatinib-resistant (1.2 µM) conditions (dashed line = 0). **C** Differential expression (log₂ fold change) of selected genes in STAT5A-knockout K562 cells under baseline and ponatinib (3 nM) conditions (dashed line = 0)

Following STAT5A knockout, RT-qPCR profiling revealed distinct yet interconnected transcriptional alterations across parental and TKI-resistant K562 models (Fig. 7A–C), underscoring the context-dependent regulatory functions of STAT5A in chronic myeloid leukemia. In parental K562 cells, deletion of STAT5A led to a pronounced repression of STAT3 (−10.61)—a central mediator of survival and proliferation—accompanied by downregulation of BAX (−0.93) and RUNX3 (−2.48), suggesting suppression of both intrinsic apoptotic triggers and tumor-suppressive transcriptional programs. In parallel, ATM was markedly upregulated (2.92), reflecting activation of DNA damage response signaling as a compensatory mechanism, while BCR (0.75) modestly increased, possibly indicative of feedback activation within BCR::ABL1–dependent circuits (Fig. 7B).

In imatinib-resistant K562 cells (1.2 µM), STAT5A loss reshaped the transcriptome toward an apoptotic and stress-responsive state, evidenced by the upregulation of TP53 (1.50), BCL-X (0.97), CASP3 (1.15), CISH (1.16), and mTOR (1.40) (Fig. 7B). These changes highlight a dual effect: (i) reactivation of p53-driven apoptotic cascades, and (ii) partial engagement of adaptive mTOR and CISH signaling, likely functioning as compensatory feedback loops within the JAK/STAT axis. Meanwhile, STAT5B (1.13) upregulation denotes a possible isoform-level compensation for STAT5A loss, although CASP8 (−1.64) and ATM (−1.95) downregulation suggests reduced extrinsic apoptotic priming and dampened DNA repair signaling under imatinib stress. In ponatinib-resistant cells (3 nM), STAT5A knockout elicited a distinctive pro-apoptotic signature characterized by significant activation of CASP8 (2.24), BCL-X (1.53), BCR (2.06), and TP53 (1.04), coupled with notable repression of STAT5B (−1.57) and JAK2 (−0.83) (Fig. 7C). These alterations collectively imply that STAT5A ablation in ponatinib-resistant cells enhances extrinsic apoptosis via CASP8 activation, while concurrently attenuating JAK2/STAT5B–mediated survival signaling, effectively disrupting compensatory resistance pathways.

Collectively, these transcriptional landscapes reveal that STAT5A acts as a master node orchestrating survival–apoptosis balance. In addition, STRING-based protein–protein interaction analysis revealed dense connectivity among *STAT5A*, *TP53*, *CASP3*, and *STAT3*, reinforcing their cooperative role in modulating apoptotic and DNA damage responses. Its disruption reprograms resistant CML cells toward apoptotic sensitivity and reduced proliferative capacity. Importantly, the magnitude and directionality of these changes are context-specific: STAT3 repression and ATM activation dominate in parental cells, TP53 and CASP3/BCL-X reactivation define the imatinib-resistant profile, and CASP8-driven extrinsic apoptosis underlies the ponatinib-resistant response. Thus, STAT5A knockout reinstates apoptotic competency through both intrinsic and extrinsic pathways, dismantling key resistance circuits and positioning STAT5A as a therapeutically actionable vulnerability in TKI-refractory CML.

### STAT5A knockout reveals key transcriptional changes in control and TKI-resistant K562 cells

In untreated K562 control cells (K562 UT), absolute expression values were generally low across most genes, except for BAX (2^-ΔCt = 0.23) and RUNX3, which showed relatively higher basal expression (Fig. 7a). Following STAT5A knockout, expression of pro-apoptotic genes BAX (0.23 → 0.12) and CASP3 (0.08 → 0.04) decreased, while CASP8 remained unchanged, suggesting a mild suppression of intrinsic apoptotic signaling. Anti-apoptotic markers BCL-X and BCL2 showed minimal variation. Meanwhile, STAT1 and STAT3 levels slightly declined, aligning with attenuation of survival signaling. DNA repair and proliferation-associated genes ATM and ABL1 were notably downregulated, whereas TP53, mTOR, and RUNX3 showed modest decreases (Figs. 6A and 7B). These data suggest that STAT5A ablation in parental K562 cells induces a restrained apoptotic response accompanied by impaired proliferative signaling.

In imatinib-resistant K562 cells (1.2 µM Ima/Res UT), baseline expression levels of STAT5A, BCL-X, and CASP3 were slightly elevated compared to control, consistent with a survival-oriented transcriptional state (Fig. 7A). Upon STAT5A knockout, expression of TP53, BCL-X, CASP3, and RUNX3 increased, reflecting partial reactivation of apoptotic and tumor-suppressive pathways (Fig. 7B and C). Concomitantly, upregulation of STAT1, mTOR, and CISH suggested activation of compensatory feedback circuits within the JAK/STAT axis, while CASP8 and ATM suppression implied reduced extrinsic apoptosis and DNA repair signaling. Collectively, these shifts indicate a dual remodeling effect: (i) reactivation of p53-driven apoptotic cascades and (ii) adaptive signaling realignment to counter imatinib-induced stress.

**Fig. 8:**
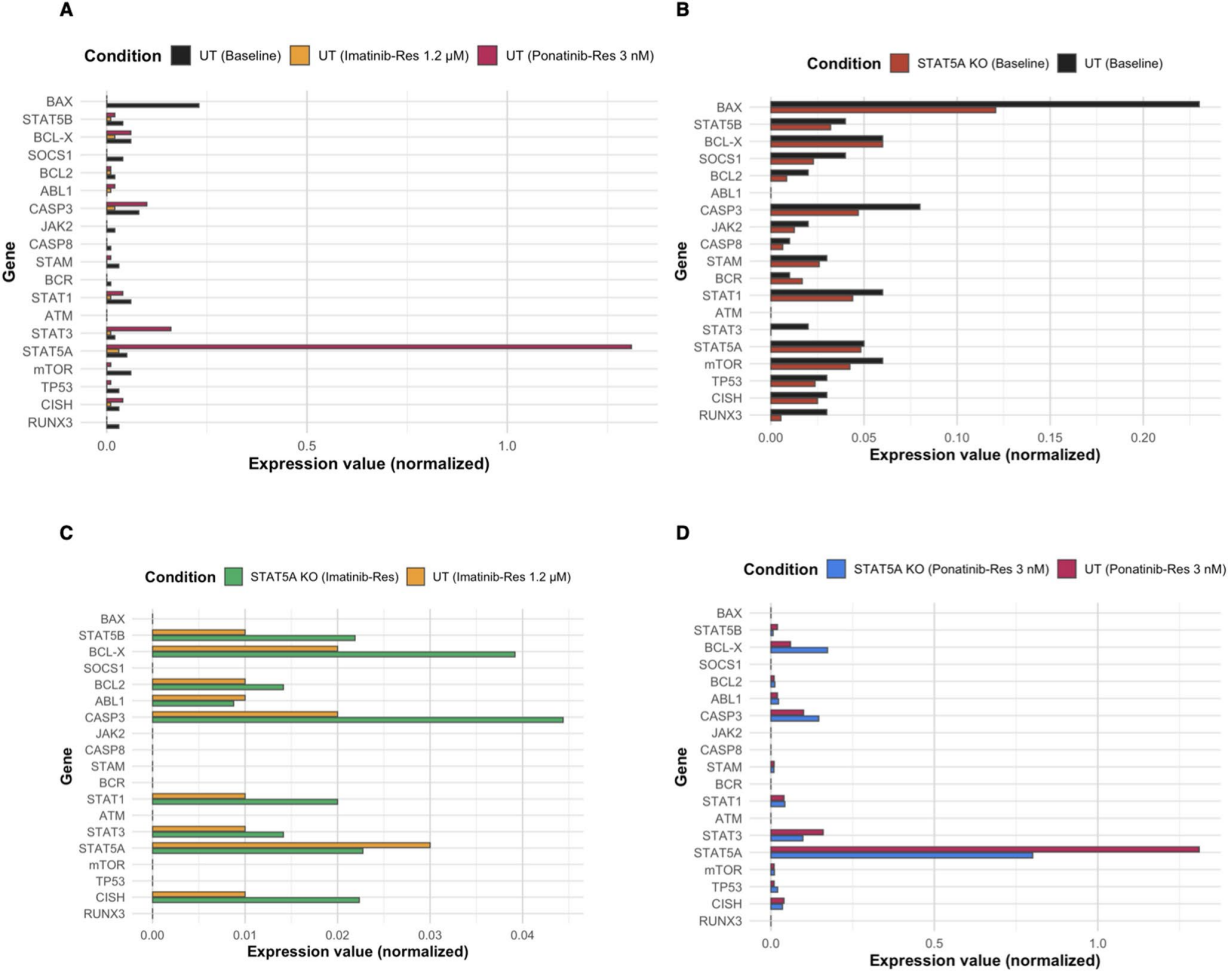
Normalized expression profiles of selected genes across baseline and TKI-resistant conditions in STAT5A-knockout and untreated K562 models. **A** Normalized expression values of selected genes across baseline, imatinib-resistant (1.2 µM), and ponatinib-resistant (3 nM) conditions in untreated K562 cells. **B** Comparison of normalized expression levels of selected genes between STAT5A-knockout K562 and untreated K562 cells under baseline conditions. **C** Comparison of normalized expression values of selected genes between STAT5A-knockout K562 (imatinib-resistant, 1.2 µM) and untreated K562 (imatinib-resistant, 1.2 µM) cells. (D) Comparison of normalized expression values of selected genes between STAT5A-knockout K562 (ponatinib-resistant, 3 nM) and untreated K562 (ponatinib-resistant, 3 nM) cells. Data represent mean ± SD from three independent experiments. Asterisks denote significance levels (*p < 0.05, **p < 0.01, ***p < 0.001, one-way ANOVA with Tukey’s post hoc test). Expression values represent relative normalized intensities derived from the corresponding UT condition using fold-change scaling (2^log2FC), and normalized values are represented as detailed in the *Materials and Methods* section

In ponatinib-resistant K562 cells (3 nM Pon/Res UT), STAT5A and STAT3 expression markedly increased (2^-ΔCt = 1.31 and + 0.83), consistent with strong survival signaling (Fig. 8A). After STAT5A deletion, a distinct transcriptional rewiring occurred—CASP8, BCL-X, and TP53 were upregulated, while STAT5B and JAK2 were repressed (Figs. 6C and 7D). These alterations highlight a context-specific apoptotic re-sensitization, in which extrinsic apoptosis via CASP8 activation is restored, and JAK2/STAT5B-mediated survival is attenuated. The concomitant downregulation of STAT3 and ATM further suggests disruption of DNA repair and proliferative control mechanisms. When integrated across all models (Figs. 7A and C and 8A and D), STAT5A knockout consistently reprogrammed transcriptional networks toward apoptotic activation and stress adaptation in a resistance-dependent manner. In parental cells, repression of STAT3 and induction of TP53/RUNX3 emphasize disruption of proliferative control and restoration of tumor-suppressive signaling. In imatinib-resistant cells, activation of CASP3, STAT1, and mTOR denotes an apoptotic shift coupled with metabolic rewiring. Finally in ponatinib-resistant cells, CASP8- and TP53-driven apoptosis was reestablished, undermining key resistance circuits. Collectively, these findings establish STAT5A as a transcriptional hub balancing survival and apoptosis. Its loss dismantles STAT3/STAT5B-dependent survival pathways, reinstates intrinsic (BAX, CASP3, TP53) and extrinsic (CASP8, BCL-X) apoptotic circuits, and restores apoptotic competency across resistant states, underscoring its therapeutic vulnerability in TKI-refractory CML.

Together, these results demonstrate that *STAT5A* depletion reprograms transcriptional and phenotypic outcomes across both parental and TKI-resistant CML models. By reinstating apoptotic competence and disrupting compensatory STAT3/STAT5B signaling, *STAT5A* knockout effectively restores TKI sensitivity. The following Discussion contextualizes these findings within existing literature and explores their translational implications for CML therapy.

## Discussion

Chronic myeloid leukemia (CML) is a hematologic malignancy driven by the BCR::ABL1 fusion gene, whose constitutively active tyrosine kinase promotes unchecked proliferation and survival [16, 17]. The BCR::ABL1 oncoprotein continuously activates mitogenic and anti-apoptotic pathways such as PI3K/AKT, RAS/MAPK, and JAK/STAT, thereby sustaining leukemic cell growth and preventing programmed cell death [18, 19]. Although tyrosine kinase inhibitors (TKIs) such as imatinib have revolutionized CML therapy and markedly improved survival [4, 20], resistance remains a critical challenge, particularly through BCR::ABL1–independent mechanisms that reactivate compensatory survival networks [5].

Signal transducers and activators of transcription (STAT) proteins are key transcription factors that convey cytokine and growth-factor signals to the nucleus, regulating proliferation, differentiation, and apoptosis [21]. Among them, STAT5A and STAT5B are constitutively activated in CML and other hematologic malignancies, functioning as essential mediators of leukemogenesis [22–24].

Casetti et al. (2013) further demonstrated that selective attenuation of STAT5A, but not STAT5B, increased oxidative stress and DNA damage in resistant CD34⁺ CML cells, highlighting its distinct role in maintaining leukemic stem-cell fitness under TKI pressure [25]. Beyond kinase signaling, persistent STAT5 activation has been recognized as a parallel survival axis that supports therapy resistance [26–28]. Pharmacologic inhibitors such as pimozide suppress STAT5 phosphorylation, impair proliferation, and induce apoptosis in resistant cells, underscoring the potential of STAT5A-focused therapeutic strategies [29]. In parallel, Nelson et al. (2011) revealed that STAT5 directly transactivates MDR1 and TERT, linking its sustained activation to multidrug resistance and prolonged leukemic cell survival [30]. Collectively, these findings position STAT5A as a multitiered regulator of chemoresistance and cellular longevity, extending its function beyond canonical BCR::ABL1 signaling.

Our data demonstrated that STAT5A and CDK4 were efficiently edited in K562, imatinib-resistant (K562/Ima1.2), and ponatinib-resistant (K562/Pon3) cell lines using the CRISPR/Cas9 system delivered via Lipofectamine CRISPRMAX. The observed editing efficiencies were consistent with previous reports; Yu et al. (2016) achieved an average indel rate of 20 ± 2% in K562 cells using the same lipid-based delivery platform [31]. In our study, STAT5A and CDK4 knockout groups exhibited comparable indel frequencies of 18.8% and 19.2%, respectively, confirming that Lipofectamine CRISPRMAX represents a feasible, reproducible, and low-toxicity method for genome editing in suspension leukemia cells. These efficiencies are also in line with subsequent lipofection-based optimization studies in hematologic cell models [32, 33].

Our findings demonstrate that CRISPR/Cas9-mediated STAT5A knockout in K562 and drug-resistant derivatives leads to a marked enhancement of apoptotic activity. In parental K562 cells, STAT5A deletion increased apoptosis by 1.9-fold, while in imatinib-resistant cells this effect was more pronounced, reaching an 11.3-fold elevation. Consistently, Kaymaz et al. (2015) reported a 3.1-fold increase in apoptosis following siRNA-mediated STAT5A silencing in imatinib-resistant K562/IMA-3⁺ cells, supporting the notion that STAT5A depletion reinstates apoptotic competence [34].

In ponatinib-resistant cells, STAT5A knockout caused a 4.1-fold increase, comparable to findings by Gumus et al. (2023) [29], who showed that pharmacologic STAT5 inhibition by pimozide induced a 14-fold apoptotic response at nanomolar concentrations. This progressive pattern across parental and resistant models suggests that STAT5A inactivation sensitizes CML cells to apoptotic stimuli in a resistance-dependent manner, in line with recent findings that sustained STAT5 signaling is required for leukemic cell survival and confers anti-apoptotic resistance [35]. To our knowledge, this represents the first CRISPR-based investigation of STAT5A loss in K562 models, underscoring the mechanistic and translational relevance of STAT5A as a survival regulator in leukemic cells.

Cell cycle analysis revealed that control K562 cells were predominantly accumulated in the G₀/G₁ phase (46.3%), consistent with previous findings [36]. Similarly, imatinib-resistant cells (41.8%) exhibited a comparable G₀/G₁ distribution, in agreement with Zhang et al. (2021) [37]. In contrast, ponatinib-resistant cells displayed 46.0% accumulation in G₀/G₁, which differs from the S-phase arrest previously reported in ponatinib-resistant K562 cells [29]. Following STAT5A knockout, both parental and resistant K562 lines exhibited a clear G₀/G₁ arrest, indicating a blockade in cell-cycle progression rather than a mere redistribution among phases. This observation is consistent with Casetti et al. (2013) [25], who described a limited yet indirect role of STAT5A attenuation in cell-cycle control, primarily mediated through weakened long-term survival signaling, and with Kollman et al. (2019), who reported G₀/G₁ arrest following STAT5A depletion [38]. Collectively, these findings indicate that STAT5A exerts minimal direct control over canonical cell-cycle checkpoints but is crucial for sustaining proliferative competence and survival under tyrosine kinase inhibition, in line with recent evidence linking the STAT5A–CDK4 signaling axis to leukemic cell-cycle regulation [39].

Detailed analysis on differences in gene expression revealed distinct transcriptional reprogramming following STAT5A knockout in K562 cells and their imatinib- and ponatinib-resistant derivatives. In ponatinib-resistant cells, CASP8 was markedly upregulated (+ 2.24-fold), indicating reactivation of apoptotic cascades and demonstrating that STAT5A targeting can trigger cell death even in resistant clones. Similarly, STAT3 expression was strongly downregulated (− 10.61-fold) in STAT5A KO K562 cells, confirming the pivotal role of STAT5A in regulating STAT3-dependent survival signaling and its contribution to maintaining TKI resistance [37, 40]. Notably, genes associated with the DNA damage response and cell-cycle regulation were also significantly altered. ATM expression increased (+ 2.92-fold), and TP53 was upregulated, reflecting reactivation of genomic surveillance and repair pathways [41, 42]. The upregulation of BAX (+ 1.45-fold) and RUNX3 further demonstrates that STAT5A depletion activates not only apoptotic but also tumor-suppressive transcriptional programs [43]. Collectively, these findings indicate that the STAT5A–STAT3–TP53–BAX axis orchestrates resistance-associated transcriptional rewiring and that STAT5A loss rebalances survival and stress-response pathways, partially restoring apoptotic sensitivity in resistant CML cells [39].

Importantly, beyond canonical STAT signaling, recent evidence has revealed a STAT5A/miR-202-5p/USP15/Caspase-6 regulatory axis that suppresses apoptosis and contributes to imatinib resistance [44], indicating that STAT5A also modulates post-transcriptional apoptotic control mechanisms. Conversely, upregulation of survival-associated genes such as BCL2, BCL-XL, and mTOR suggests activation of compensatory pathways, implying that STAT5A targeting alone may be insufficient to fully overcome resistance [24, 45]. Together with our bioinformatic analysis, these molecular findings highlight that STAT5A depletion triggers multi-layered transcriptional adaptations that integrate apoptotic, cell-cycle, and survival networks. Collectively, these findings indicate that STAT5A orchestrates interconnected gene networks governing survival, apoptosis, and metabolic signaling, underscoring the potential benefit of combinatorial therapeutic approaches targeting the STAT5A axis together with parallel resistance pathways.

While *STAT5A* inhibition alone reinstates apoptotic signaling, residual activation of compensatory survival pathways such as PI3K/AKT and mTOR suggests that combination strategies may yield superior therapeutic benefit. This observation aligns with recent evidence indicating that dual blockade of STAT5A and PI3K pathways synergistically overcomes TKI resistance in advanced CML [45]. Future studies integrating multi-target inhibition are warranted to evaluate this combinatorial efficacy.

Overall, our findings demonstrate that STAT5A orchestrates apoptotic, pro-survival, and tumor-suppressive pathways in CML cells, thereby maintaining TKI resistance. These results are consistent with earlier reports identifying STAT5A as a critical survival factor and potential therapeutic target in hematologic malignancies [46, 47] and are further supported by recent evidence highlighting the STAT5A–CDK4 and STAT5A–PI3K axes as key drivers of therapy resistance [39, 45]. Future studies should focus on functional validation of STAT5A-targeted strategies in patient-derived xenografts or primary CD34⁺ CML samples to confirm their translational applicability and clinical feasibility.

## Conclusion

This study provides functional and mechanistic evidence that STAT5A acts as one of the master regulators of survival and apoptotic signaling in chronic myeloid leukemia (CML), sustaining resistance to tyrosine kinase inhibitors (TKIs). Using CRISPR/Cas9-mediated knockout, we demonstrated that loss of STAT5A reprograms aberrant JAK/STAT and DNA-damage response pathways, leading to activation of intrinsic (TP53, CASP3, ATM) and extrinsic (CASP8, BCL2L1) apoptotic circuits and restoration of chemosensitivity. These findings define STAT5A as a mechanistically validated and therapeutically actionable target in TKI-refractory CML. Future translational efforts integrating STAT5A inhibition—through CRISPR-based functional targeting or small-molecule inhibitors such as pimozide—with standard TKIs may provide a rational combinatorial strategy to overcome resistance and achieve durable clinical responses.

## Supplementary Information

Below is the link to the electronic supplementary material.

Supplementary Material 1

Supplementary Material 2

## Data Availability

No datasets were generated or analysed during the current study.
