# Supplementary material for: Targeting STAT5A via CRISPR/Cas9 restores TKI sensitivity in resistant chronic myeloid leukemia cells
Source: Med Oncol. 2026 Apr 25;43(6):184. doi: 10.1007/s12032-026-03295-6 (PMC13110212; doi:10.1007/s12032-026-03295-6)
Supplement: Supplementary file 1 — Supplementary Material 1 [file 12032_2026_3295_MOESM1_ESM.docx]

**Supplementary Table 1. Primer Sequences and Gene Information Used for Quantitative Real-Time PCR (qRT-PCR)**

| **BAX :** | F:5’-ATCATGGGCTGGACATTGG-3’  R:5’-TGGAGACAGGGACATCAGT-3’ |
| --- | --- |
| **BCR** | F: 5’GCTTCCGCATGATCTACCTG-3’  R: 5’CTGGCCTTACCCGGAGAAC-3’ |
| **ATM:** | F:5’GCTCAGGAAGGAATGAGAGAAA-3’  R:5’CCACAGCTATCAACGTCAGTAA-3’ |
| **STAM:** | F:5’CCCAGGTGCCAAACTATAACT-3’  R:5’AGCAGAGCCTTCTCAGAATATG-3’ |
| ***BCL2L1 (BCL-xL*** | F:5’-TCAGGCTGCTTGGGATAAAG-3’  R: 5’-AGGCTTCTGGAGGACATTTG-3’ |
| ***TP53*** | F: 5’-AGGGATGTTTGGGAGATGTAAG- 3’  R:5’-CCTGGTTAGTACGGTGAAGTG-3’ |
| ***BCL-2*** | F: 5’-GTGGATGACTGAGTACCTGAAC-3’  R :5’-GAGACAGCCAGGAGAAATCAA-3’ |
| ***MTOR*** | F: 5’GGGACAGCATGGAAGAATACA-3’  R:5’TGTTGTGCCAAGGAGAAGAG-3’ |
| ***RUNX3*** | F:5’AGGCGTAAGGGAACTCATAAAG-3’  R:5’CTCTCACAGAGACAACCAATGA-3’ |
| ***ABL1*** | F: 5’AAGCCGCTCGTTGGAACTC-3’  R: 5’AGACCCGGAGCTTTTCACCT-3’ |
| ***CISH*** | F:5’GCCACTGCTGTACACCTAAA-3’  R:5’ACCAGACGGTTGATGACAAG-3’ |
| ***STAT1*** | F:5’-GACGAGAATGAGGGTCCTTTG-3’  R: 5’-CTACGTCAAGCAGTTCCCTAAA-3’ |
| ***CASP3*** | F: 5’-GCTGCCTGTAACTTGAGAGTAG-3’  R: 5’-GTATGGAGAAATGGGCTGTAGG-3’ |
| ***CASP8*** | F:5’-GGAGCTGCTCTTCCGAATTA-3’  R:5’-CATGACCCTGTAGGCAGAAA-3’ |
| ***STAT3*** | F: 5’-GGAGCAGAGATGTGGGAATG-3’  R:5’-GTGATACACCTCGGTCTCAAAG-3’ |
| ***STAT5A*** | F*:*5’GATAGGTAGGGCATGGGCAAGG-3’  R: 5’CTGGATGGTAGGGACCCTCT-3’ |
| ***STAT5B*** | F:5’CCGCTTGGGAGACTTGAATTA-3’  R:5’CAGGGAACTGGTGTGTAGTA-3’ |
| ***SOCS1*** | F: 5’CACGCACTTCCGCACATTC-3’  R:5’ TAAGGGCGAAAAAGCAGTTCC-3’ |
| ***JAK2*** | F: 5’TCTGGGGAGTATGTTGCAGAA-3’  R: 5’AGACATGGTTGGGTGGATACC-3’ |
| ***GAPDH*** | F: 5’CAAGAGCACAAGAGGAAGAGAG-3’  R:5’CTACATGGCAACTGTGAGGAG-3’ |
|  |  |
