# Supplementary material for: Targeting STAT5A via CRISPR/Cas9 restores TKI sensitivity in resistant chronic myeloid leukemia cells
Source: Med Oncol. 2026 Apr 25;43(6):184. doi: 10.1007/s12032-026-03295-6 (PMC13110212; doi:10.1007/s12032-026-03295-6)
Supplement: Supplementary file 2 — Supplementary Material 2 [file 12032_2026_3295_MOESM2_ESM.docx]

**Supplementary Figure S1. Full, uncropped gel image corresponding to Figure 3C.**


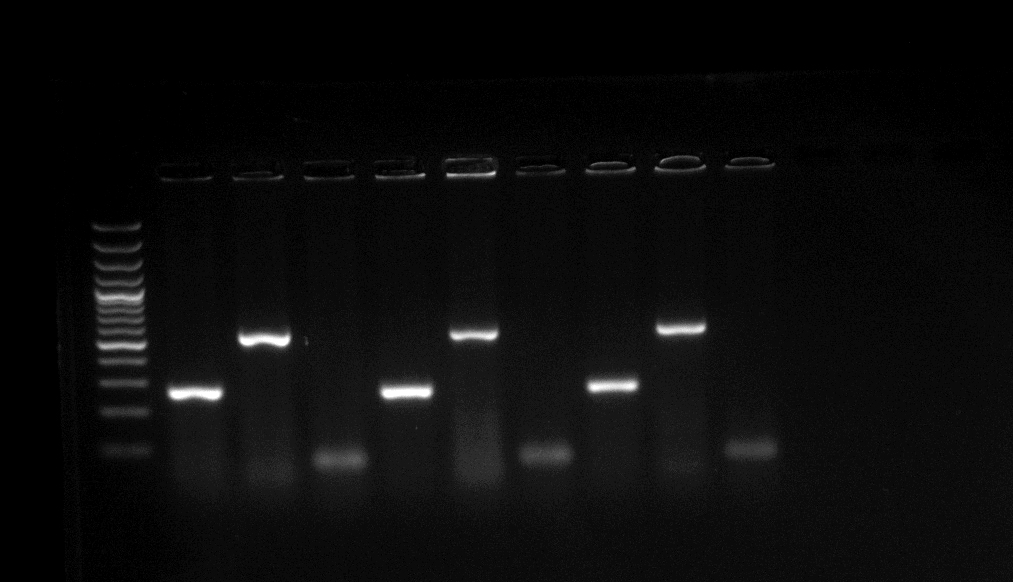


**Supplementary Figure S2. Full, uncropped gel image corresponding to Figure 3D.**

**
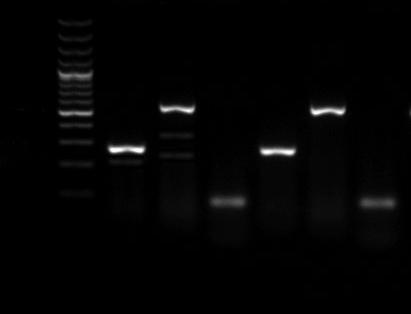
**
